# The evolving role of multi-modality imaging in transcatheter tricuspid valve interventions

**DOI:** 10.3389/fcvm.2022.793267

**Published:** 2022-10-25

**Authors:** Livia Luciana Gheorghe, Romy Hegeman, Martijn Vrijkorte, Nina Wunderlich, Joao Cavalcante, Dee Dee Wang, Bushra Rana, Mani Vannan, Leo Timmers, Martin Swaans

**Affiliations:** ^1^Interventional Cardiology, Hospital Universitario Puerta del Mar, Cádiz, Spain; ^2^Cardiovascular Imaging Department, St. Antonius Hospital, Nieuwegein, Netherlands; ^3^Cardiovascular Imaging Department, Asklepios Kliniken Langen-Seligenstadt GmbH, Röntgenstrasse 20, Langen, Germany; ^4^Cardiovascular Imaging Department, Allina Health, Minneapolis, MN, United States; ^5^Cardiovascular Imaging Department, Henry Ford Health System, Detroit, MI, United States; ^6^Cardiovascular Imaging Department, Imperial College London, London, United Kingdom; ^7^Piedmont HealthCare, Statesville, NC, United States

**Keywords:** imaging, echo, MDCT (multidetector cardiac computed tomography), structural heart intervencions, tricuspid valve, percutaneous intervention

## Abstract

Tricuspid valve pathophysiology is not well-understood. Emergence of novel transcatheter tricuspid therapies has fueled the requirements for improved imaging visualization techniques and interventional imaging physician skillsets in guiding these complex transcatheter procedures. There is growing understanding on the clinical significance of tricuspid regurgitation which expanded the interest for percutaneous tricuspid valve interventions. The present review concentrates on three essential aspects of tricuspid valve pathophysiology: anatomical considerations for tricuspid interventions, optimal timing of tricuspid interventions by imaging guidance, and the role of interventional imaging physicians’ skillset and knowledge in this field.

## Introduction

Tricuspid regurgitation (TR) natural history is not well understood and presents challenges to clinical practice. The majority of severe TR is secondary due to annular dilatation resulting from right ventricular (RV) disease or less commonly right atrial (RA) dilatation. Severe TR if left untreated eventually results in right heart failure (RHF). Irrespective of the cause, TR is associated with worse survival regardless of left ventricular (LV) function and pulmonary hypertension (1). Indeed, late residual TR seen post mitral valve surgery (2) and isolated severe TR (3) are associated with excess mortality and morbidity. This heterogeneous patient group includes right ventricular (RV) dysfunction, pulmonary hypertension and multiple associated comorbid conditions. Surgical TV intervention studies have suggested variable outcomes and this conflicting data have led to confusion as to optimal management. As a result, patients with severe TR are often referred late for intervention reflecting a high perioperative mortality rate approx. 8-10% (4, 5).

As the heart team evolves, the growing experience of transcatheter therapies has highlighted the dilemma of optimal management of severe TR. In patients undergoing either surgical or transcatheter mitral or aortic valve interventions, severe TR is an important marker of poorer prognosis at both short- and long-term follow-up. The present era of structural heart transcatheter interventions brings to the forefront the importance of precisely defining the pathophysiology. The interventional imaging team now assumes a central role. To this end this review aims to highlight the evolving key concepts in the application of multimodality imaging in the assessment, patient selection and procedural guidance in patients undergoing transcatheter tricuspid valve interventions.

## Tricuspid valve morphology assessment

The tricuspid valve is traditionally described consisting of three leaflets, anterior (A), posterior (P) and septal (S), which are thin translucent structures, no more than a few millimeters in thickness. The septal leaflet is shortest in length (also known as height, i.e., in the radial direction) and least mobile. Importantly, recent 3D TEE imaging assessment has highlighted the significant variability (46%) in the number of functional leaflets (6). Four types are described; Type I is the typical three leaflets (54%); Type II is a bicuspid valve (A and P fused, 5%); Type III where one leaflet has two functional parts, A (3%), P (32%), S (4%) making a 4 leaflets valve; Type IV has two functional leaflets seen in both A and P (2%) leaflets making a 5 leaflets valve (Table 1). Clearly identify the number of functional leaflets is essential when assessing suitability for transcatheter edge to edge therapy (TEER). Where the leaflets meet is known as the *coaptation zone*. The coaptation length is approximately 5-10 mm, which maintains valve competency. Progressive leaflet tethering, prolapse or annular dilatation results in malcoaptation with a reduction in coaptation length. This is the basis for the mechanism of regurgitation and if severe can result in frank loss of coaptation zone resulting in a *coaptation gap*. Identification of a coaptation gap is pathognomonic of severe regurgitation. Landmarks are key to correct orientation and identification of valve leaflets. The coronary sinus is located near the postero-septal commissure. The antero-septal commissure is situated near the aortic valve/root (points toward the commissure between non-and right coronary cusps) and its location helps identify the position of the anterior leaflet. The ventricular/atrial septum identifies the septal leaflet. Lastly identifying the anterior papillary muscle (often joining with the moderator band) assists in locating the antero-posterior commissure (6, 7). In terms of transcatheter therapies, two structures in close proximity may be at risk of damage. The right coronary artery (RCA) which runs adjacent to the muscular portion of the TV annulus, for approximately two-thirds of its circumference, in the right atrioventricular groove. The atrioventricular node and conduction system is located just below the insertion point of the septal leaflet in the membranous portion of the ventricular septum (7). A detailed assessment of the co-aptation zone and mechanism of TR can be done by integrating multiple 2D imaging views. 3D imaging allows precise analysis using multiplane reconstruction. This method permits on-axis alignment with the coaptation zone, where a section-by-section interrogation of the coaptation zone (between the antero-septal, postero-septal and antero-posterior co-aptation zones and centrally where the three leaflets meet) leaflet motion, thickness, calcification as well as subvalvular apparatus and tricuspid annulus (TA) anatomy can be performed. Applying the principles of Carpentier’s classification, mechanism of leaflet failure can be identified. The TV orifice is approximately 9 cm^2^, where owing to the lower pressure chambers typically has a mean TV gradient of no more that 2 mmHg (8).

**TABLE 1:** A new proposal for a standard echocardiographic tricuspid valve nomenclature.

Type of valve configuration	Leaflets number	Leaflet’s name
Type I	3-leaflet configuration	A/P/S
Type II	2-leaflet configuration	A-P/S
Type III	4-leaflet configuration	
IIIA		A1/A2/P/S
IIIB		A/P1/P2/S
IIIC		A/P/S1/S2
Type IV	5-leaflet configuration	A1/A2/P1/P2/S

A: anterior; P: posterior; S: septal.

TV subvalvular apparatus consists of 2-3 papillary muscles (PM). Anterior, the largest gives rise to chords to the anterior and posterior leaflets. It is positioned below the antero-posterior commissure (being attached to the lateral wall) and serves as a useful landmark and may join the moderator band. The posterior PM typically consists of 2-3 heads with chords to the posterior and septal leaflets. Lastly the septal PM may not always be present (absent in 20% of normal population) and instead replaced by chords from the septal or anterior leaflet directly inserting into the ventricular septum (7).

The tricuspid annulus (TA) is D shaped. The septal leaflet inserts to the atrial/ventricular septum forming the more linear section with variable degrees of fibrous tissue. While the remainder C shaped muscular portion is demarcated at the transition between atrial and ventricular myocardium where anterior and posterior leaflets insert. The normal TA circumference is approximately 12 ± 1 cm, and area 11 ± 2cm^2^ (7, 9). Forming a dynamic non-planar shape with a high point at its antero-septal portion (more atrial) and low point at the postero-septal region (more ventricular), it can change in size by up to 30%, being largest during end systole and early diastole. In disease states the TA dilates with relative sparing along its septal leaflet attachment and being greatest in its inferior portion (border of posterior leaflet attachment). Important differences are seen in TA geometric alterations in different disease states in secondary TR. In pulmonary hypertension (PH) the RV tends to elongate (increase in RV length) relative to its base resulting in a less spherical RV geometry. This results in displacement of the PM producing greater tethering of the leaflets towards the RV (with less annular dilatation) producing a larger tenting height/area. While in idiopathic TR (heterogeneous group without PH and including patients with AF) a greater annular dilatation rather than leaflet tethering is seen (10). Established cut-off values taken from surgical data for optimal timing of intervention include TA > 40 mm or 21 mm/m^2^ (RV focused four chamber view in end diastole) (11). Unfavorable outcomes with residual TR post-surgical repair are seen where tenting height >0.76 cm and tenting area >1.63 cm^2^ (12). 3D annular sizing (circumference and area) is possible using multiplanar reconstruction with both TTE and TEE datasets, similar to CT.

## Grading of tricuspid regurgitation

**FIGURE 1:**
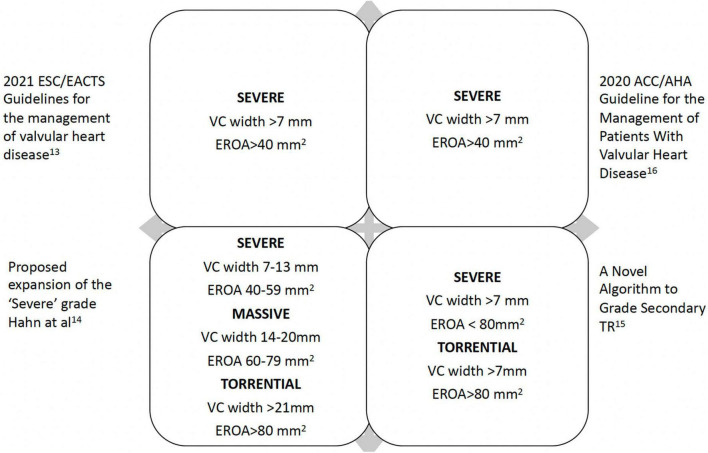
Tricuspid regurgitation grading.

Traditional severity grading has focused on color Doppler regurgitant jet imaging. Reliance on jet size and expansion within the right atrium (RA) is limited by a number of technical and hemodynamic factors. Proximal iso-velocity surface area (PISA) method (also known as flow convergence method) allows quantitative assessment, where severe TR is defined as an effective regurgitant orifice (EROA)≥40 mm^2^ with a regurgitant volume ≥45 ml (13). TR defined by the EROA cut-off ≥ 40 mm^2^ has been shown to be associated with excess mortality and morbidity during 10-year follow up (3). Evaluation of TR severity requires a holistic approach and should be combined with supportive evidence for severe regurgitation (Figure 1) (7, 13–16). Functional TR, by far the commonest, has an elliptical rather than circular regurgitant orifice morphology. Hence standard parameters are more likely to underestimate severity. Therefore, vena contracta width is the recommended parameter in TR severity grading, where ≥ 7 mm is indicative of severe TR (17, 18) and should be averaged from measurements taken in two orthogonal planes (typically apical 4-chamber/2-chamber and long-axis parasternal RV inflow and short axis parasternal inflow-outflow views). While a number of quantitative parameters exist (using stroke volume calculations) newer 3D techniques such as vena contracta area (VCA) (19) are likely to offer improved diagnostic accuracy since they allow precise alignment with the TR jet with correct plane orientation to measure the optimal vena contracta dimensions (true short and long axis) and area. However, in primary TR, it is much more difficult to measure VCA since the annulus is a curvilinear structure, and the jet direction may come from different coaptation line at one time and not permit correct alignment.

In the era of transcatheter tricuspid valve therapies, it has become evident standard 2D methods underestimate the true severity of TR (20). A new grading system, utilizing 3D vena contracta dimension and area as additional parameters, has been proposed with three grades within the ‘severe’ category (severe, massive and torrential cut-off values for VC (biplane) 7, 14, 21mm and VCA 75, 95 and 115 mm^2^, respectively) (14, 21). A recent application of this concept has demonstrated prognostic significance using a VC cut-off 14 mm (22). Importantly integrating TR supportive parameters (8) including hepatic vein systolic flow reversal, right atrial dimensions and volume, along with RV parameters and pulmonary pressure estimation (where possible) is essential both for pre and post-procedure follow-up. Lastly during assessment, it should be remembered the hemodynamic setting can alter the findings and a note should be made of the blood pressure, volume status in the context of heart failure medications, as well as serial changes in right ventricular (RV) function.

## Assessment of the hemodynamic consequences of tricuspid regurgitation

The right ventricle is sensitive to both pressure and volume changes. Quantifying changes in RV size and function are essential in determining the stage of disease. An enlarging RV carries worsening prognosis in a number of disease states both pre and post-surgery (23–27). Several RV parameters can be assessed and have been described previously (28). Importantly, the recommended measure is RV volumes and RV ejection fraction (RVEF) currently providing the most useful prognostic parameter. While data is sparse, limited studies suggest indexed RV end diastolic volumes > 150 ml/m^2^ may carry a worse outcome (24, 25), and where RV function is severely impaired, RVEF < 30% may represent prohibitive risks for conventional surgery and potentially end-stage disease. Currently cardiac magnetic resonance (CMR) is the reference method but is relatively expensive and not widely available (RVEF men 62% (± 5 normal range 52–72), women 61% (± 6 normal range 51–71) (29). 3D echo (3DE) has good correlation with CMR in RV volumes and RVEF calculation. 3DE, techniques are improving and increasingly used in Echo Labs. Despite a systematic underestimation of volumes (although not ejection fraction) of approximately 20% by 3DE (likely reflecting limitations in endocardial border detection and incomplete capture of the entire RV within the 3DE window), studies comparing 3DE with CMR have shown good correlation (30, 31). The semi-automated border detection method is recommended (32) with current cut-off values normal range 58% ± 6.6, where abnormal <45% (women systolic, diastolic volumes 27 ± 8.5 mL/m^2^, 61 ± 13 mL/m^2^, men 22 ± 7 mL/m^2^, 53 ± 10.5 mL/m^2^). RV global longitudinal strain provides important insights into RV performance, offering useful indicators of prognosis and functional capacity in a number of disease states (33, 34). It has been shown to correlate with myocardial fibrosis, a marker of poor prognosis (35) and may play a role in risk stratification and timing of intervention. Values lower –20% (or more negative) are considered normal and while RV function remains preserved would be expected to be in this range in the setting of severe TR (28).

## Assessment of pulmonary hypertension: Importance of right heart catheterization

When determining the appropriateness and optimal timing for TV intervention, careful assessment of any underlying increased pulmonary vascular resistance (PVR) and associated pulmonary disease (such as pulmonary fibrosis or chronic obstructive airways disease) is necessary. Further differentiating between isolated post-capillary PH (IpcPH) or later stages with combined post-and pre-capillary PH (CpcPH) in the setting of chronic left heart disease is important. Particularly where the latter may have progressed to irreversible obstructive and fixed remodeling of the pulmonary arteries such that the relief of severe TR may have little positive impact on RV outcome (28). Non-invasive assessment of PH has limitations when TR is massive or torrential. Echo diagnosis is dependent on a reliable pressure difference between the RA and RV in systole. The TR continuous wave Doppler (CWD) profile becomes triangular with a low peak velocity since there is rapid equalization of pressures between the two chambers. However, the estimated pulmonary artery pressure depends not only on the value of RV/RA gradient, but also of the right atrium size and pressure, which is semi-quantitative evaluated by the size of the inferior vena cava. Nevertheless, in some patients the RA pressure may reach 30-40 mmHg (with ventricularization of the right atrium) which is not reflected during the echo measurement. Supportive parameters including pulmonary acceleration time (< 100 msec), pulmonary regurgitation CWD early-diastolic and end-diastolic gradients may give clues to the presence of increased pulmonary vascular resistance (PVR), but do not differentiate type or extent. Further, progressive RV failure results in a reduction in flow velocities as the RV is unable to generate high pressures despite a high afterload/PVR. This can result in a low TR velocity and hence non-invasive measure of PH is not enough to define its presence. Therefore, there is a role for right heart catheterization to elucidate the presence, severity, and etiology of PH and especially in those cases where are signs of pulmonary hypertension, but the values are not concordant, the measurement of the pulmonary vascular resistance is crucial. The key parameters have been described previously (28). Involvement of PH experts may be required.

## Etiology of tricuspid regurgitation

An understanding of the mechanisms for TR is essential, since this directs (36) treatment options. TR can occur as a result of primary valve disease or more often be secondary to annular dilatation usually from right heart chamber enlargement, typically RV dilatation and dysfunction. However, progressive right atrial dilatation in the setting of atrial fibrillation may result in secondary TR (36). Deciphering the underlying etiology and integrating the relation of the RV, pulmonary circulation, and any related comorbid conditions is key to determining best treatment options. Key patient groups include primary TR where a range of etiologies may cause primary leaflet disease. Clues to the possible etiology may be evident following a careful review of the patient’s history (i.e., previous blunt trauma to the chest, myocardial biopsy or radiotherapy treatment) and clinical examination (i.e., autoimmune disorders). Identifying leaflet abnormalities including prolapse with chordal rupture or leaflet thickening with retraction and chordal shortening may support the diagnosis.

In the setting of left heart (LH) valve disease, the majority of TR is functional in origin. Despite a fall in RV afterload, it does not predictably improve following correction of the LH valve lesion. Surgical correction for severe TR at the time of LH valve disease is performed routinely (13). Further, less than severe TR may progress over months to years to severe symptomatic TR following LH valve surgery with an associated poor survival (2). Unfortunately finding significant TR late after LH valve surgery is not uncommon, reported as 8% to as much as 49% (37–39). Although RV dysfunction was shown to be the best predictor of outcome during long-term follow-up after LH valve surgery (40), the presence of TR and its severity is associated with significantly worse morbidity and mortality (38, 39). Following surgery, the TV annulus may continue to dilate despite the improvement and/or absence of pulmonary hypertension where age, presence of AF and left atrial dilatation are predictors of significant late TR. Published data highlights high operative mortality often due to late referral. Perioperative mortality usually results from right heart failure and hepatorenal syndrome, progressing to multi-organ failure and death. Careful risk stratification before considering corrective TV surgery should include not only RV function status and pulmonary vascular hemodynamic assessment, but also the consequences of chronic systemic venous hypertension resulting in congestion and hypoperfusion (28). Optimizing medical therapy may improve both RV function and TR severity. Those with significant RV dilatation and impairment and/or fixed pulmonary hypertension, along with hypersplenism (low hemoglobin and platelets counts), renal and liver failure (raised bilirubin, abnormal clotting), despite optimal medical therapy, often reflect end-stage disease, where TV intervention is likely to be futile (28, 41).

In patients with cardiac implantable electronic device (CEID), the incidence of TR has been reported between 6 and 10% (1, 42, 43). CEID lead interference leading to TR can be categorized into three types: TV chordal apparatus entanglement, septal leaflet impingement and leaflet perforation. A chronic endocardial fibrotic reaction at the site of lead contact may lead to encapsulation, ensheathment or entrapment. Tethering of the leaflets and subvalvular apparatus may further exacerbate TV dysfunction and progressive TR. Detail imaging is essential and 3D echo and multiplane reconstruction formats are particularly useful to ascertain the relationship of the RV lead with the TV leaflets and subvalve apparatus. Assessment of RV function should include signs of RV dysynchrony. If image quality and expertise allow, this can be obtained using 3DE. Cardiac MRI (CMR) is increasingly utilized as devices become CMR compatible. Where neither modality is possible cardiac computerized tomography (CCT) may be a useful alternative. Pulmonary hypertension is not usually a feature in this patient group unless there is LH disease. Treatment options may include minimizing pacing burden (in the case of brady-pacing) or lead repositioning with preferential His-bundle pacing. The aim being to improve RV dysfunction and subsequently TR severity through reduction in RV dysynchrony. Where these are not an option then surgical lead explantation and TV repair (43, 44) may be considered. Lastly, TR may also result from trauma during lead extraction or lead-related infection.

Isolated functional TR is associated with RA enlargement in the setting of chronic atrial fibrillation (45). RA remodeling has been shown to result in TV annular dilatation. This may result in loss of annular leaflet coverage reserve without leaflet tethering. Interestingly, the geometric changes subsequent to primary RA dilatation result in is more circular and planar TV annulus which is more dilated when compared to functional TR due to RV dilatation (from LH disease) in sinus rhythm. Further, in the case of functional TR secondary to AF, TV annular area is strongly associated with RA volumes, unlike LH-associated TR which principally correlates with RV volumes. In terms of management, addressing heart rhythm and rate control as well as ventricular function through optimizing medical therapy is the first line. RA remodeling may result in reduction in TR severity. Similar to when pacing settings are optimized, chamber remodeling may take several months. Further re-evaluation is usually done 3-6 months later.

Progressive pulmonary arterial hypertension (PAH) is associated with RV dilatation and dysfunction underlies the development of progressive secondary TR. Treatment is focused on identifying the cause of PAH and its management. Where appropriate medical therapy with pulmonary vasodilators aims to reduce RV afterload. As RV function improves, reduction in TR severity follows. Where severe TR persists, it usually reflects progressive RV disease, where residual TR may paradoxically work to off-load the failing RV. Surgical intervention is rarely considered and reserved for carefully selected patients undergoing specific disease modifying treatments. This includes pulmonary thromboendarterectomy, where the expected reduction in PAH and RV afterload will support RV remodeling and any concurrent TV intervention (28).

## Tricuspid regurgitation treatment: Right moment: Right therapy

### Medical therapy

**FIGURE 2:**
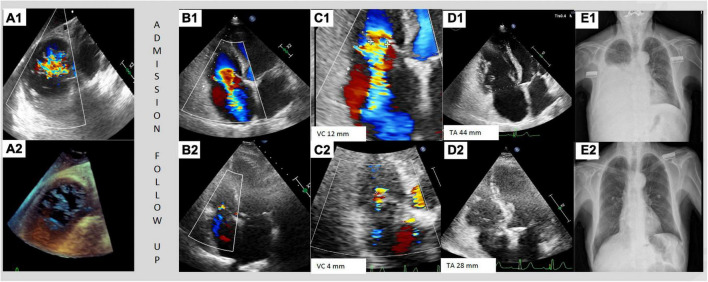
Case of dynamic changes of TR during medical therapy. Patient with previous aortic surgical replacement admitted for right heart failure and torrential tricuspid regurgitation. **(A1, A2)** Show the transesophageal transgastric view with torrential tricuspid regurgitation and no-coaptation between anterior and septal leaflet. **(B1–E1)** Images during the admission and (**B2–E2)** images during the follow-up with intensive diuretic treatment. The transthoracic echocardiography showed a torrential TR with a jet which filled the entire atrium **(B1)**, vena contracta of 12 mm **(C1)** and a tricuspid annulus of 44 mm **(D1)**. The thoracic radiography at admission with serious right pleural effusion **(E1)**. Three months follow-up transthoracic echocardiography showed a mild TR **(B2)** with a vena contracta of 4 mm **(C2)** and a regression of tricuspid annulus size of 28 mm **(D2)**. The thoracic radiography at follow-up showed no pleural effusion.

Optimal management of severe TR initially involves treatment of the underlying causative disease process (see above) with concomitant HF treatment. Medical therapy with diuretics is currently the basis treatment for severe TR with right heart failure (RHF). With progression of TR, high doses or a combination of different diuretic groups is often necessary to control RHF. Concomitant neurohormonal antagonist administration can have an additional effect on diuretic therapy by reducing the negative effect of compensating mechanisms triggered by volume loss. When significant systemic venous congestion is present (liver derangement, coagulation disorders, renal failure, or edema of the bowels) oral diuretics may be ineffective, and intravenous therapy or in selected cases peritoneal dialysis or hemofiltration with addition of inotropic support may be necessary (46). Response to diuretic therapy is related to outcomes in patients with acute heart failure (47). Serial imaging during medical treatment is important, since intensive diuretic therapies when implemented early in the diagnosis of RHF may improve the degree of TR and trigger reverse RV remodeling and reduction in tricuspid annular (TA) size (47). An example is shown in Figure 2. However, where targeted therapy does not impact positively on TR severity and RV function then surgical intervention, where appropriate, should not be delayed or, in the case of inoperable disease, transcatheter options such as edge to edge repair considered. Since symptom control is not associated with disease control and progressive RV impairment carries a poor outcome.

### Surgical treatment (repair or replacement)

Surgical intervention can be considered for both primary and secondary TR. Most often this is performed as a concurrent procedure at the time of left heart surgery. Class I indication for TV intervention is recommended for severe TR (whether primary or secondary) at the time of left heart (LH) valve surgery in both American (16) and ESC/EACTS guidelines (13) or in isolated severe primary TR without severe RV dysfunction. TV surgery should be considered (Class 2a indication) in the following circumstances: in those with *primary severe* TR who are mildly (or asymptomatic) with RV dilatation and are suitable for conventional surgery; *primary moderate* TR undergoing left-side valve surgery. For *secondary* TR isolated surgery should be considered in symptomatic patients with severe TR (with or without previous LH valve surgery) in the absence of severe RV or LV-dysfunction or severe pulmonary vascular disease/hypertension; or in those with mild or moderate TR undergoing left heart valve surgery who have a dilated TV annulus. TR progression after LH valve surgery is a predictor of mortality (due to RHF) and, for this reason moderate TR or annulus dilation (>40 mm or > 21 mm/m^2^) should be treated during surgery. Reoperation may be an option in patients with severe secondary TR in the absence of severe pulmonary hypertension or RV systolic dysfunction. However, there is a substantial patient population beyond this narrow definition with symptomatic TR refractory to medical therapy.

Surgical TV repair involves TV annuloplasty. The primary goal is to improve leaflet coaptation by correcting annular dilatation and restoring annular geometry (48). Incomplete rigid or semirigid undersized rings are typically used to avoid damage to the conduction system and AV node. When leaflet tethering is present, performing TV annuloplasty alone is often inadequate and concomitant leaflet repair techniques are required (e.g., leaflet augmentation, “clover” suture, or double orifice valve technique). In the presence of echocardiographic risk factors for TR recurrence after TV annuloplasty (larger annular diameter, advanced leaflet tethering, reduced LV function <40%) (48), the Heart team may recommend surgical tricuspid prosthesis implantation, most often a bioprosthesis with long-term (if tolerated) oral anticoagulation.

### Transcatheter interventions

The role of transcatheter interventions for TR stems from the unmet need of large patient populations with symptomatic TR deemed unsuitable for conventional surgery. Much like early TAVR device evolution, current TR technologies are in their infancy. Currently four tricuspid transcatheter technologies are CE marked. Using early generation devices Tamarasso et al. (49) showed improved clinical outcomes using transcatheter therapy compared with medical therapy alone in patients with severe TR, deemed high surgical. Review of the current scientific data and experience has led to TV transcatheter therapies being a Class IIb recommendation in the current 2021 ESC/EACTS guidelines for inoperable patients with severe secondary TR (13).

There are many conceptual ways to tackle right heart failure secondary to TR. Novel technologies based on surgical strategies of repair and annuloplasty have been developed via a transcatheter approach (50–53). Out of the box concepts in treating secondary symptoms of severe TR have resulted in development of heterotopic tricuspid valve replacement (54). In a heterotopic valve replacement, stent valves may be implanted in the inferior vena cava, or simultaneously in the superior and inferior vena cavae to relieve symptoms in patients with clinically significant tricuspid regurgitation. The four transcatheter tricuspid devices with CE mark for treatment of TR are: the Triclip (Abbott Vascular, Santa Clara, Califorina, United States) and the Pascal system (Pascal and Pascal ACE, Edwards Lifesciences, Edwards Lifesciences, Irvine, California, United States), both leaflet edge to edge repair (TEER) techniques; the Cardioband device (Edwards Lifesciences, Irvine, California, United States) for tricuspid annuloplasty; the TricValve (P + F Products + Features Vertriebs GmbH, Vienna, Austria) device which is a heterotopic valve for patients with end-stage TR, a transcatheter bicaval valves system preventing caval reflux.

## Role of multi-modality imaging in case selection and procedure guidance

### Role of echocardiography

Echocardiography is the main pillar in each step of TR evaluation spanning diagnosis planning, procedural guidance, and follow-up assessment (Figures 2A1-D1, 3). TV morphology and TR severity evaluation using TTE has been extensively described by Hahn et al. (8). TEE confirms the diagnosis and allows a more accurate evaluation of the mechanism(s) contributing to each patient’s underlying disease state. Detailed leaflet segmental analysis and TV annulus geometry and sizing parameters (tenting height and area and the tenting volume) can be assessed using 3D echo, as well as an appreciation of surrounding anatomy. TEE protocol should include mid-esophageal views sweeping through multiple angles (0°, 45°, 90°, 135°) to assess leaflet anatomy and TR severity. Transgastric short axis view allows an en face view of leaflets, number, functional components, location of the origin of TR along the coaptation line, coaptation gap and areas of leaflet prolapse, restriction or leaflet retraction and calcification. Specific considerations for types of devices used in transcatheter TV therapies are described below.

**FIGURE 3:**
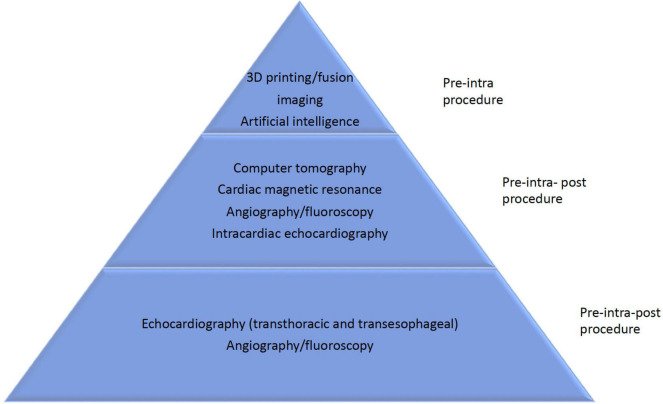
Imaging tools for tricuspid valve.

Transcatheter leaflet edge-to-edge repair (TEER): The aim of TEER technique is to target the antero-septal coaptation line starting close to the commissure and moving centrally (“Zip” technique). Some parameters have been described as predictors of unfavorable results after tricuspid TEER including: a septo-lateral coaptation gap > 7 mm (55); TV EROA > 0.6 cm^2^; tenting area > 3.2 cm^2^ (56); and TR vena contracta width > 11 mm (57). Optimal candidates for TEER approach have 3-leaflet morphology, with normal leaflet mobility (particularly of the septal leaflet) with limited tethering (tricuspid leaflet coaptation gap < 8.4 mm) (58), and absence of dense chordal structures and with no anatomical restrictions for the inferior vena cava (IVC) –right atrium (RA) approach.

**FIGURE 4:**
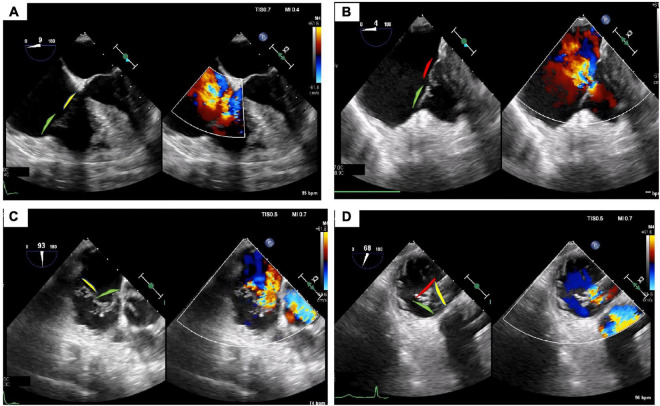
Tricuspid regurgitation treated with 3 TriClips implantation. Transesophageal evaluation of the tricuspid regurgitation (2D/color). **(A)** 2D mid-esophageal view at 0° showing the anterior and septal leaflet. **(B)** Deep-esophageal view 0° showing the jet between anterior and posterior leaflet. **(C)** Mid-esophageal view at 90° showing the jet between the anterior and septal; **(D)** transgastric view short axis, in systole, the valve is closed, and the jet is mainly located between the anterior and septal leaflet. Anterior leaflet: green; Septal leaflet: yellow; Posterior leaflet: red.

**FIGURE 5:**
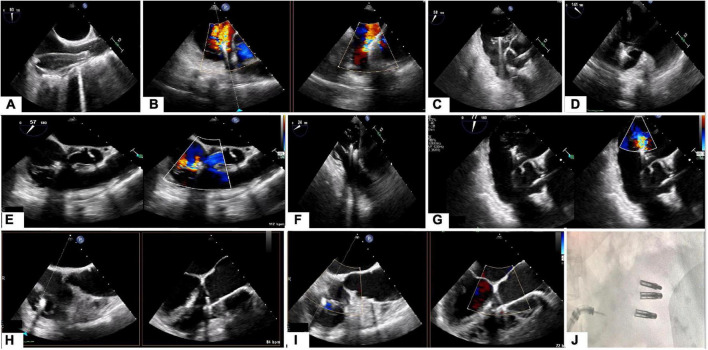
Triclip procedure: **(A)** bicaval view, delivery catheter advancement. **(B)** Deep esophageal 60° and X-plane view (150°) color showing the first clip in the middle of the main regurgitant jet. **(C)** 2D mid esophageal view at 60° with the clip localized between anterior and septal leaflet **(D)** 2D deep esophageal view at 140°, the clip opened at 180°. **(E)** Mid-esophageal view at 90° showing first clip implanted and residual tricuspid regurgitation. **(F)** Transgastric view, placing second clip at anterior and septal leaflet. **(G)** Mid esophageal view at 80° (2D and color) showing the results after second clip implantation with a residual jet between anterior and posterior leaflet. **(F)** 2D enface view (and X-plane) with the tricuspid valve opened and a third clip located between anterior and posterior leaflet. **(I)** Color enface view (and X-plane) of the tricuspid valve with trace tricuspid regurgitation. **(J)** Angiographic view with the presence of 3 implanted clips.

**FIGURE 6:**
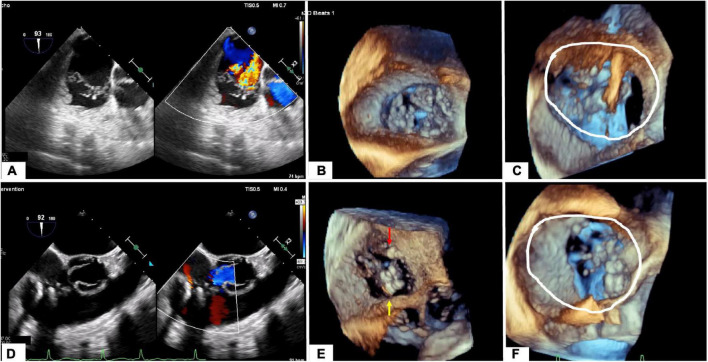
Tricuspid regurgitation before **(A–C)** and after procedure **(D–F)**. **(A)** Mid-esophageal view at 90° showing the main jet between the anterior and septal. **(B)** Transgastric enface view 3D showing a gap between anterior and septal leaflet and anterior and posterior leaflet. **(C)** Annular dimension before first clip implantation. **(D)** Mid-esophageal view at 90° showing the final result with 3 implanted clips and trace tricuspid regurgitation. **(E)** Transgastric enface view 3D showing the localization of the clips: 2 clips between anterior and septal leaflet (yellow arrow) and 1 between anterior and posterior leaflet (red arrow). **(F)** Reduction of annular dimensions after clips implantation.

For the TEER technique, the device is aligned perpendicular to the tricuspid leaflet coaptation line (mainly between anterior and septal leaflets) using 2D, transgastric, multi-plane, and 3D enface views of the TV. The advancement of the delivery systems, and TEER device may be performed using 2D mid-esophageal biplane view (inflow-outflow view), the same view used during leaflet grasping/clasping. Application of the 3D enface view and transgastric short axis views demonstrate the position of the device along the coaptation line and allow for additional assessment of correct leaflet grasping. Upon completion of grasping, color Doppler and continuous wave Doppler flow velocities are obtained to evaluate for any significant change in TV gradient as well as residual TR. To date there have not been quantitative parameters validated to assess the results after tricuspid TEER technique. The system removal is also performed under TEE guidance using the bicaval view and 3D view from the RA to avoid right atrial wall damage. An illustrative case is presented in the Figures 4–6.

Annuloplasty repair therapies (ART): The objective of transcatheter annuloplasty using the Cardioband device, is to imitate a surgical annuloplasty band (i.e., incomplete annuloplasty ring). Anchors runs counter-clockwise from the antero-septal to the postero-septal commissure (i.e., the coronary sinus) to prevent damage to the conduction system in the trigonum. Intraprocedural imaging with TEE allows visualization of the delivery catheter, the device navigation at the level of the right atrium, device coaxiality and final TR assessment.

Some anatomical relative contraindications should be taken into consideration for transcatheter tricuspid ART since the presence of very eccentric/lead induced regurgitant, excessive annulus dilatation, leaflet tethering or pseudo-prolapse may lead to a suboptimal result.

Heterotopic transcatheter valve replacement (HTVR): Although fluoroscopy is the main imaging modality in HTVR procedures (transcatheter bicaval valves system), TEE can be helpful in assessing the correct valve orientation in orthotopic procedures, the position of the leaflets on the anchors (i.e., EVOQUE valve), and to evaluate the results. For HTVR the key is to visualize the superior vena cava (SVC) and inferior vena cava (IVC), identify the landmarks and the possible interference with the hepatic veins or valve displacement.

#### Follow-up device evaluation

TTE is the main imaging tool for follow-up evaluation. The results of transcatheter tricuspid intervention are evaluated at key intervals, 30 days, 6 months, and at 1 and 2 years. Assessment of residual or recurrent TR is evaluated using the standard multi-parameter approach similar to pre-procedure and includes a combination of EROA (derived from the volumetric and PISA methods), VC width (biplane) and VCA, and annular dimensions evaluated in two orthogonal views (septo-lateral and antero-posterior). Serial evaluation in right heart chamber size, volumes, function are important parameters and help determine response to therapy and outcomes.

The presence of a tricuspid TEER device (TriClip/Pascal device) make assessment TR severity more difficult post-intervention. The 30-day post procedure TTE is generally used to evaluate both TR severity and correct device position (complications such as leaflet device detachment typically arises shortly after the procedure). Early data suggest potential decrease in annular dilatation at 1 to 2 years post intervention (50, 53).

### Computer tomography

Comprehensive computed tomography (CT) may overcome many of the limitations from 2D echocardiography by providing excellent anatomical and functional analysis of the tricuspid valve apparatus and its relation to the nearby structures. CT can provide additional information allowing assessment of the anatomical regurgitant orifice area (59). The latter is especially useful in the presence of a mechanical aortic prosthesis where acoustic shadowing limits precise visualization during echocardiography. Functional CT provides complimentary data to TEE when determining suitability for TEER vs. TV replacement, including an understanding of TV leaflets, location, and size of any coaptation gap and potential interaction of the leaflet with pacemaker/ICD leads. Moreover, analysis of venous connections and right heart structures may help determine and optimize the requirements of future valves and delivery systems (size, length, and flexibility). CT is not required for TEER procedures, but it is fundamental for transcatheter TV replacement (heterotopic/orthotopic valves) and annuloplasty devices.

**FIGURE 7:**
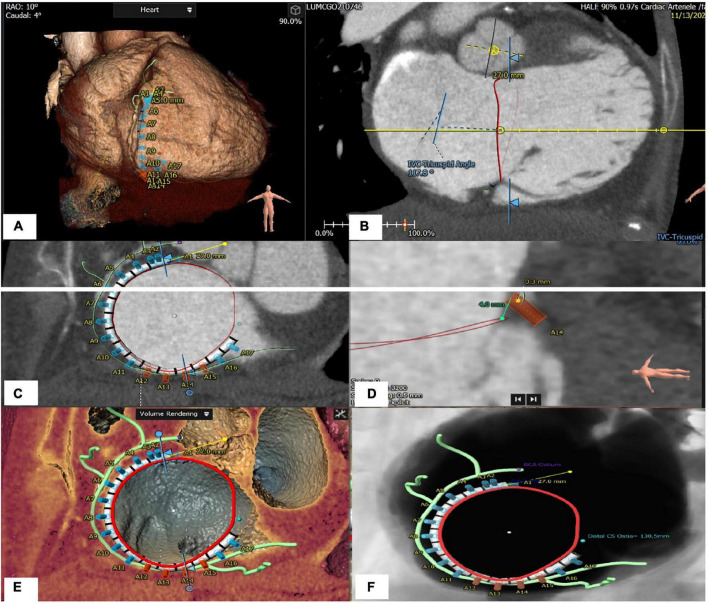
Tricuspid apparatus analysis of Cardioband procedure. **(A)** Projection of the virtual Cardioband device at the level of TA. **(B)** Measurement of the angle between TA and VCI (128°). **(C)** Enface view of TA showing a simulation of the Cardioband implantation (17 anchors). **(D)** Measurement of the distance between the closest anchor (A14) to the RCA. **(E)** Enface view of TA with the Cardioband device and trajectory of the RCA. **(F)** Measurement of the distance between the last anchor and coronary sinus (13 mm).

A precise sizing of the TA and its alteration during the cardiac cycle is necessary to ensure suitability for current device (Cardioband, replacement valves). The Cardioband device can not cover a TA circumference >120 mm, and orthotopic valve a TA diameter >52 mm (where valve oversizing will be < 10%.). The preferred modality is CCT since it allows accurate multi-planar annular reconstruction (Figure 7). While echocardiography may approximate the 2D diameter of the TA (septo-lateral diameter), high quality multi-beat 3D-TEE acquisition of the TV and then MPR allow the measurement of the diameter, perimeter of the TA and the curvilinear distance between the antero-septal and postero-septal commissure. It is important to understand the tricuspid anatomy with respect to its surrounding anatomical structures. During Cardioband implantation a risk of right coronary artery (RCA) injury due to the proximity may exist. Distances less than <2 mm between the TA and the RCA risk vessel injury by annuloplasty anchoring mechanisms. The RCA course is generally closer to the anterior leaflet insertion than the posterior leaflet insertion. In patients with significant TR, an unfavorable course of the right coronary artery may be present in up to 28% at the level of the anterior leaflet and 13% at the level of the posterior leaflet (60). Key anatomical parameters include TA diameters, perimeters and area, RV dimensions, IVS-TA angles. In addition, the ability to identify a number of potential landing zones allow for optimal procedural planning. Unlike transcatheter mitral valve implantation, where outflow tract obstruction is a concern, the risk of RV outflow tract obstruction is minimal due to the anatomical relationship and angle between the outflow and inflow of the RV.

CT plays a central role in suitability and procedure planning for heterotopic caval devices including parameters such as precise distance between surrounding structures; SVC diameter at the level of the innominate vein confluence and SVC-RA junction; IVC at the level of the junction with the RA; distance between RA and hepatic vein orifice(s) and their relationship with the IVC-RA junction (should be > 10 mm to avoid hepatic vein obstruction).

Finally, CT can determine optimal fluoroscopic projections for guiding the procedures, essential in helping to reduce procedural times and radiation exposure. Four main fluoroscopic projections have been described which may be superimposed on to the principal echocardiographic views and help both interventional imaging and implanting team with fusion imaging guidance (Table 2).

**TABLE 2:** Correlation between CT/fluoroscopy projections and TEE echocardiographic views.

CT projection	Echo view	Details
LAO/CAU (55°/15°)	1-chamber enface view	Tricuspid valve Coronary artery course Coronary sinus
RAO/CAU (60°/50°)	2-chamber view	Anterior and posterior leaflet of the TR and subvalvular apparatus
RAO/CRA (25°/15°)	3-chamber view	Posterior and septal leaflet of the TR RV outflow tract
LAO/CRA (50°/60°)	4-chamber view	Anterior and septal leaflets of the TR

CAU: caudal; CRA: cranial; CT: computer tomography; LAO: left anterior oblique; RAO right anterior oblique; RV: right ventricle; TR: tricuspid regurgitation.

Specific CT acquisition protocols focusing on the right-side are employed for tricuspid imaging. In general, CT scanners with a higher number of detectors enables shorter breath-hold, lower radiation and contrast utilization. In the setting of atrial fibrillation, CT scanners offering higher temporal resolution such as the Siemens dual-source CT scanners (preferably b80 ms) can improve image quality (61). In summary, CCT imaging plays a central role in procedure planning particularly in annuloplasty and valve replacement therapies. It may also play a role during follow-up examinations where doubts exist regarding tricuspid apparatus/device evaluated with the first line imaging modalities (TTE/TEE).

### Cardiac magnetic resonance

**FIGURE 8:**
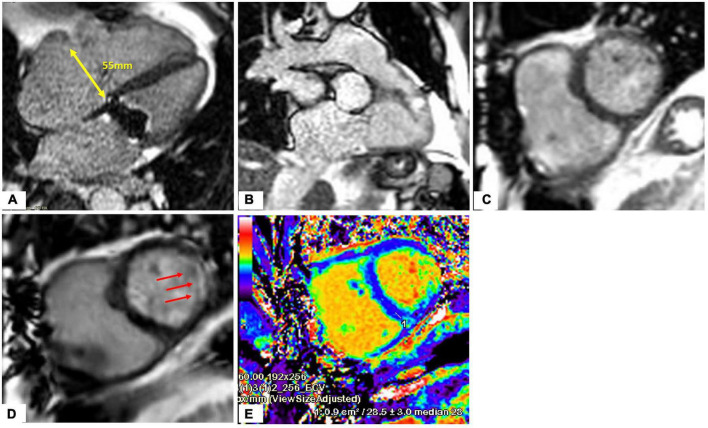
Heart evaluation in patient with significant TR and prior mechanical MV replacement using CMR. **(A–C)** Cine imaging of several views of the RA/RV. **(D)** Late gadolinium enhancement imaging show basal lateral infarct (red arrows). **(E)** Extracellular volume fraction (ECV) mapping showing upper normal value for LV septum and in the corresponding lateral area which matches the replacement fibrosis seen on **(D)**, significantly elevated supportive of prior infarct.

**FIGURE 9:**
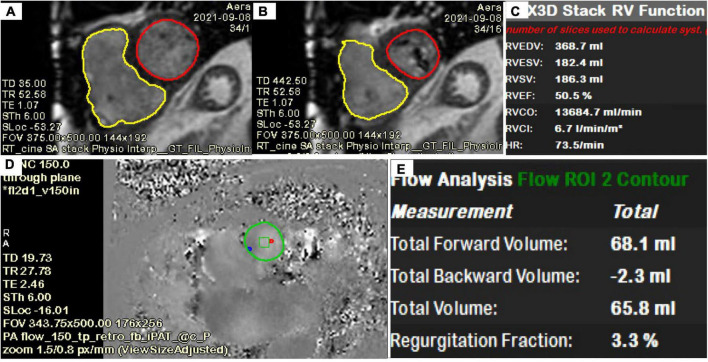
Quantification of LV, RV, TR volume using CMR. **(A–C)** Quantification of biventricular volumes, ejection fraction and stroke volume using semi-automated tracing of short-axis cine. **(D,E)** 2D phase contrast quantifying forward flow through main pulmonary artery. The delta (RVSV-PA flow) is the TR volume (118 ml). TR fraction (63%) is the TRVol/RVSV.

Cardiac magnetic resonance (CMR) provides an opportunity to assess RV dimensions, volumes, RV ejection fraction, quantification of TR and non-invasive calculation of pulmonary vascular resistance (PVR). Pulmonary hypertension (PH) is one of the Achille’s heels of tricuspid treatment and is usually diagnosed late because of the non-specificity of the symptoms. RV performance and adaptation to an increased afterload, reflects the interaction of the pulmonary artery (PA) and RV as a morphofunctional unit (PV-PA coupling) (62). In situations of end stage right ventricular failure, the pulmonary artery pressure may be lower, but the PVR increased (63). The measurement of the PVR using CMR is feasible and may help improve the selection of candidates for tricuspid interventions (63). Distinct CMR acquisitions are useful for a more complete evaluation of the right heart: (1) Cine steady-state free-precession (SSFP) and phase-contrast regional area change have been suggested as powerful biomarkers for prognosis and treatment; (2) Strain analysis with myocardial feature tracking is useful to detect early RV dysfunction; (3) 2D phase contrast quantifies forward flow and allows for indirect assessment of TR severity, which has prognostic value (64); (4) late gadolinium enhancement (LGE) imaging detects the presence of infarction, infiltrative processes and non-ischemic fibrosis seen in PH patients (Figures 8, 9). Promising CMR techniques in this population are the use of extracellular volume mapping for the quantification of interstitial fibrosis, and of 4D flow to allow simultaneous quantification of multivalvular disorders.

Multiple challenges exist for obtaining quality imaging studies during CMR acquisition in structural heart patients. Variability in heart rates in the setting of atrial fibrillation may prevent accurate ejection fraction quantification in a gated cardiac MRI sequence. Presence of intracardiac devices such as older generation pacemakers/ICDs may create artifacts, limiting chamber evaluation. Lastly, in the setting of acute heart failure, patients may not be able to tolerate the duration of the MRI study or number of breath-holds required for each image stack acquisition. Technological CMR developments such as single-breath hold or complete free-breathing imaging acquisition (65, 66), in the most vulnerable patients with atrial arrhythmias are actively being investigated and validated for valvular heart studies (65).

### Intracardiac echocardiography

Intracardiac echocardiography (ICE) was developed as a potential alternative to TEE mitigating the need for a general anesthetic in its use during structural heart disease interventions. Two available phased-array systems are AcuNav V (Siemens-Acuson, Mountain View, California) and ViewFlex Xtra (Abbott Vascular, St. Paul, Minnesota). There are 5 “standardized” positions for ICE probe imaging (mid RA, low RA, RV inflow tract, RV outflow tract, and left atrium) (66). ICE allows for the visualization of larger devices to help guide the navigation and coaxiality inside right heart chambers (67).

Several proof-of-concept reports have supported the use of ICE in guiding transcatheter TV annuloplasty, leaflet repair, valvuloplasty, and TV-in valve implantation (68, 69). Although ICE may be a promising imaging tool there are some non-negligible limitations: limited experience among operators, the imperfection of the current probes (limited far-field imaging, 3D capabilities and lack of biplane imaging), imaging artifacts and interference with the implanted device (for example heterotopic valves), and high costs of each single-use device.

### Fusion imaging

**FIGURE 10:**
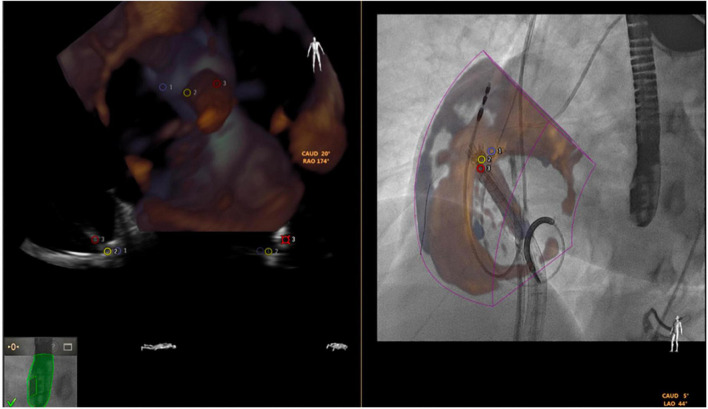
Fusion of 3-dimensional echocardiography and fluoroscopy (using EchoNavigator) in a transcatheter tricuspid Cardioband direct annuloplasty.

The fusion imaging systems combine different imaging tools (e.g., CT or echocardiography) with fluoroscopy, creating image overlay on a single screen. The Philips EchoNavigator (Philips Healthcare) and Siemens Syngo TrueFusion (Siemens Healthcare) software both require compatible same brand echocardiography and fluoroscopy equipment along with specialized hardware and software needed to process and create hybrid images. Limitations of current fusion technology include the color volume overlay on fluoroscopic monitors. *Static fusion imaging* (roadmapping) uses 3D data-sets acquired prior to the procedure (most commonly CT) which are combined with intraprocedural fluoroscopy. During transcatheter valve implantation it may be useful to superimpose vena cavae onto the fluoroscopic image. However, deviations in pre-procedural imaging, patient positioning, malalignment with fluoroscopic images, registration errors, or cardiac cycle selection may affect the accuracy of the fusion display and associated procedural outcomes. *Dynamic fusion imaging* has several advantages due the combination of real-time imaging data (real time 3D TEE and fluoroscopy). It facilitates greater accuracy when positioning devices within the heart. Key advantages included the ability to crop (to remove soft tissue details not relevant for the procedure), adjustment overlay translucency (to prevent fluoroscopy from being obscured) and the placing of persistent anatomic reference markers in fluoroscopic space. For tricuspid interventions such as Cardioband implantation, the fusion between the 3D TEE and fluoroscopy may give a better spatial orientation when deploying the anchors (Figure 10, courtesy of Pascual et al. (70)).

.

## Conclusion

Treatment options for tricuspid regurgitation are now rapidly expanding as transcatheter tricuspid technologies continue to evolve. An appreciation of heterogenous patient cohort and the complex interaction with the right ventricle, pulmonary circulation and left heart disease is crucial in correctly identifying those who might benefit from intervention. Where conventional surgery is deemed high risk, transcatheter therapies may play a role. Multi-modality imaging in patient selection and device choice has assumed center stage for such procedures and as such demand expert skills from the imaging team. Echocardiography remains the first-line and main imaging modality and its widespread use facilitates diagnosis. An in-depth understanding of the physiopathology of tricuspid regurgitation and its mechanisms is critical to ensuring best outcomes. It is important to highlight that accurate and timely diagnosis, optimal heart team decision making, procedural success and long-term outcomes are firmly rooted in the expertise and experience of the interventional imaging team. Hence, central to the optimal treatment of TR will be the need to value the role of the interventional imaging physician, who is equally invested in each patient’s care side-by-side with the implanting physician in this entire process.

## Author contributions

All authors contributed to the article, read, and approved this manuscript.
